# Trade-offs in antibody repertoires to complex antigens

**DOI:** 10.1098/rstb.2014.0245

**Published:** 2015-09-05

**Authors:** Lauren M. Childs, Edward B. Baskerville, Sarah Cobey

**Affiliations:** 1 Center for Communicable Disease Dynamics, Harvard T.H. Chan School of Public Health, Boston, MA, USA; 2 Department of Epidemiology, Harvard T.H. Chan School of Public Health, Boston, MA, USA; 3 Ecology and Evolution, University of Chicago, Chicago, IL, USA

**Keywords:** antibody repertoire evolution, affinity maturation, NK model, germinal centre reaction

## Abstract

Pathogens vary in their antigenic complexity. While some pathogens such as measles present a few relatively invariant targets to the immune system, others such as malaria display considerable antigenic diversity. How the immune response copes in the presence of multiple antigens, and whether a trade-off exists between the breadth and efficacy of antibody (Ab)-mediated immune responses, are unsolved problems. We present a theoretical model of affinity maturation of B-cell receptors (BCRs) during a primary infection and examine how variation in the number of accessible antigenic sites alters the Ab repertoire. Naive B cells with randomly generated receptor sequences initiate the germinal centre (GC) reaction. The binding affinity of a BCR to an antigen is quantified via a genotype–phenotype map, based on a random energy landscape, that combines local and distant interactions between residues. In the presence of numerous antigens or epitopes, B-cell clones with different specificities compete for stimulation during rounds of mutation within GCs. We find that the availability of many epitopes reduces the affinity and relative breadth of the Ab repertoire. Despite the stochasticity of somatic hypermutation, patterns of immunodominance are strongly shaped by chance selection of naive B cells with specificities for particular epitopes. Our model provides a mechanistic basis for the diversity of Ab repertoires and the evolutionary advantage of antigenically complex pathogens.

## 1. Introduction

Antibodies are an important form of protection against many pathogens, and pathogens have evolved diverse strategies to minimize their impact. For example, influenza and HIV rapidly evolve their immune targets through de novo point mutations [1,2], hepatitis B virus produces decoy particles to redirect the antibody (Ab) response [3], and malaria rapidly cycles surface proteins during an infection [4–8]. The ways in which pathogens compromise the development of effective Ab responses shape the course of infection, epidemiological patterns and the evolutionary success of different pathogen groups [9,10]. These mechanisms are relevant to vaccine strategy, as they imply that the number of antigens and the history of exposure influence Ab evolution.

Although affinity maturation consistently produces high-affinity B-cell clones, there is no general theory for the observed diversity of Ab repertoires. Naive hosts, challenged with antigen, form antibodies against a variety of epitopes. For some antigens, the majority of induced B cells within and across hosts target the same epitope, indicating consistent patterns of immunodominance [11]. For other antigens, evolved B-cell populations show adaptation to different epitopes [12–14]. These differences may arise from variability in the accessibility of binding sites on the antigen, the genetic diversity of a host's naive B-cell population, stochastic founding events in GCs, genetic predisposition mediated by helper T cells and chance mutations during affinity maturation. The relative contributions of these factors are unknown.

The primary aim of this study is to evaluate the mechanisms shaping the evolution of Ab repertoires during a single infection with an antigenically complex pathogen. The major features of our model are its explicit representations of the genetic and phenotypic diversity of B-cell populations, stochastic evolution of multiple B-cell clones and multiple scales of competitive dynamics. We examine how the number of epitopes presented by a pathogen (or other immunogen) affects the adaptation and diversity of B-cell populations, which ultimately influence protection. The model provides a parsimonious, mechanistic explanation of how different patterns of Ab repertoire diversity can arise from a few features. These simple dynamics also predict conditions under which antigenically variable pathogens compromise affinity maturation, potentially explaining the success of these pathogens. Our secondary aim is to introduce an open-source computational model of B-cell repertoire evolution as a tool to test theory.

### (a) Dynamics of affinity maturation and memory

The evolution of high-affinity antibodies occurs in germinal centres (GCs) [15,16]. Several days after an infection starts, naive B cells migrate to lymph nodes and other sites, where they aggregate with helper T cells to form GCs. Inside the GCs, B cells undergo cycles of replication with somatic hypermutation and selection. Somatic hypermutation preferentially introduces point mutations into the variable region of the B-cell receptor (BCR), the portion that interacts with antigen. On average, one mutation is introduced per round of replication [17]. The mutated B cells then compete for antigen presented by the follicular dendritic cells (FDCs) and for positive signals from local helper T cells. Higher affinity B cells in every round have a higher probability of receiving T cell help and undergoing replication, leaving lower affinity B cells to apoptose [16]. The GC reaction often terminates after a few dozen rounds of somatic hypermutation but may last for several weeks [18]. At the end of the GC reaction, average B-cell affinities have increased by several orders of magnitude [19]. This Darwinian process is known as affinity maturation.

During affinity maturation, some B cells emigrate from the GC as plasmablasts or memory cells. Emerging plasmablasts eventually differentiate into short-lived or long-lived plasma cells. Most plasma cells are short-lived and secrete antibodies, the soluble form of the BCR, at a high rate for only a few weeks [20], whereas long-lived plasma cells migrate to the bone marrow and secrete antibodies at low concentrations for years [21,22]. Memory cells may persist indefinitely, but they do not secrete antibodies [23]. Memory cells help initiate a quick response upon exposure to similar antigens. The processes regulating the switching of GC-associated B cells to memory or plasma cells are not well understood [16], although plasma cells generally have higher affinities than memory B cells [24].

Until recently, it was thought that affinity maturation occurred independently in separate GCs, with selection for increased affinity driven by local competition for antigen and T cell help [16]. Recent research shows that evolution may occur in a more coordinated manner across a metapopulation of GCs [25]. Antibodies produced by B cells from the current and previous infections compete with BCRs for antigen [26]. These antibodies are secreted systemically and may help drive affinity maturation across GCs [26].

### (b) Previous models

Mathematical models have long shed light on the competitive dynamics of affinity maturation [27–40]. Competition between B cells was originally thought to be solely for antigen [27,28], implying that small amounts of antigen should intensify competition and lead to higher affinity antibodies. Recently, models and experiments have shown that competition for antigen and support from helper T cells both influence the rate of affinity maturation [16,36,37]. It has also been proposed that competition between BCRs and antibodies may be a stronger driver of affinity maturation than competition between endogenous B cells for antigen and T cell help [26].

There has been comparatively little investigation of the competitive dynamics of affinity maturation in the presence of complex antigens. Chaudhury *et al.* [39] modelled multiple strains each with multiple epitopes that were conserved to varying degrees across strains. Cross-reactive antibodies arose to more conserved epitopes, despite higher immunogenicity of variable epitopes, supporting the idea that the growth of B-cell populations is limited by resource (antigen) availability. Increasing the number of strains and antigenic variation increased selection for antibodies that cross-reacted with variable and conserved epitopes. Wang *et al.* [40] modelled HIV-like antigens composed of a single epitope containing variable and conserved residues and assumed all epitopes were equally immunogenic. Under different vaccination strategies, including simultaneous and sequential exposure to original and mutated epitopes, affinity maturation was frequently found to be ‘frustrated’, with B cells unable to evolve high affinity to some epitopes. Broadly cross-reactive antibodies rarely evolved except under sequential immunization protocols. Under all vaccination strategies, the antibodies' breadth and affinity remained sensitive to the antigen concentration, the number of presented antigens and epitope masking.

A major uncertainty in models of affinity maturation is the impact of mutations on B-cell fitness. Fitness is commonly measured as binding affinity between the BCR and antigen. Shape-space models [41] use the sizes of B-cell- and antigen-binding regions, the polarities of their amino acids, and other physical characteristics of the B cells and antigens to define the locations and volumes of antigen and Ab in an abstract space. Typically, affinity maturation in these models entails incremental changes in these parameters, which move the Ab closer to or further from the antigen. In a similar vein, other models use metrics based on the Hamming distance, i.e. the number of unique sites in two sequences [36,39]. This formulation limits the impact of any single mutation on fitness and again favours gradual changes in affinity. The shape-space and distance-based models imply a rosy view of evolution, in that they allow monotonic increases to maximum affinity from any starting location.

A contrasting approach is the random energy landscape [42–49], originally introduced as a spin glass model. Random energy landscapes assume a stochastic mapping of genotype to phenotype. These landscapes are ‘tunably rugged’, as varying a single parameter changes the probability that a random mutation has a large or small effect. This variation in the impact of a mutation is the hallmark of epistasis, which occurs when a mutation in one genetic background has a different effect in another. Evolution thus proceeds in these landscapes not only through gradual changes in phenotype (e.g. gradual increases in affinity) but also through sudden jumps. When ruggedness is high, adaptation can lead populations to a local fitness maximum and then stop unless multiple, simultaneous mutations allow populations to traverse local fitness minima. Because epistasis and constrained adaptation appear fundamental features of protein evolution [50], we use this model to represent the molecular evolution of affinity maturation.

## 2. Material and methods

**Figure 1.**
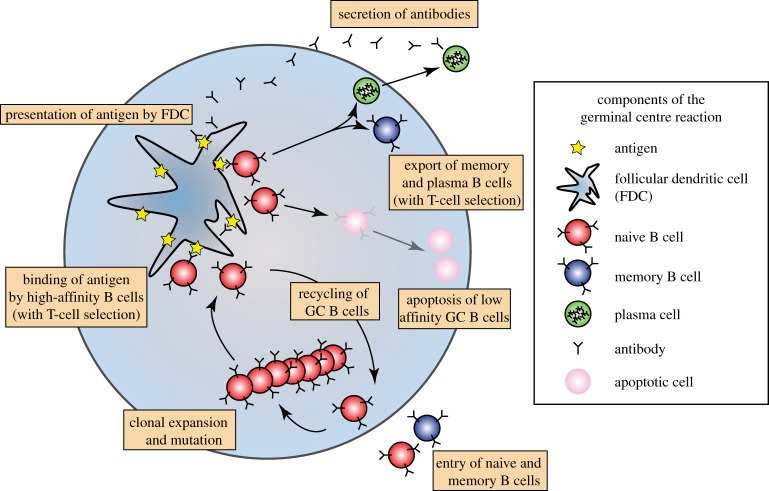
Schematic of a GC reaction. Affinity maturation of B cells occurs in the GC. Naive (or memory) B cells enter the GC and proliferate with mutation. Following proliferation, they migrate to a region containing FDCs, which present antigen. If the B cells successfully compete for antigen on the FDCs and receive positive signals from helper T cells, they either undergo additional rounds of proliferation or leave the GC as memory or plasma cells. Cells that cannot successfully compete for antigen and T cell help are lost via apoptosis. Cells that have exited the GC as plasma cells secrete antibodies that can re-enter the GC and compete with B cells for antigen, through the masking of epitopes.

We modify a classic random energy model [42–45], the NK-type model of affinity maturation introduced by Kauffman & Weinberger [46] in 1989 and extended by Deem and co-workers [47–49]. Our model incorporates aspects of the GC reaction, namely epitope masking by antibodies and cycles of proliferation and selection, hypothesized to affect dynamics [26,29]. In contrast to previous models [39,40,51], ours simulates stochastic evolution on a rugged fitness landscape, affinity to more than one epitope, and simultaneous evolution in multiple GCs. Our affinity function is uncomplicated, ignoring potential modular substructures [46–48]. We use this landscape to investigate the evolutionary dynamics of multiple competing B-cell lineages with potentially divergent specificities (figure 1).

### (a) Antigens and affinities

An antigen is described by a set of *q* epitopes, and B cells are defined by a sequence of length *L*, a proxy for the length of the Ab variable region [52]. B cells include naive cells, GC-associated cells, memory cells and plasma cells, the last of which secrete antibodies with identical affinity to the BCR. A B-cell sequence **x** maps to a vector **F** of affinities, one for each epitope of a given antigen. The affinity *F_j_* of a B cell for each epitope *j* is calculated from the epitope's random energy landscape. This landscape is a unique property of each epitope and maps a B-cell sequence **x** to an energy *U_j_*(**x**), which is the normalized sum of individual energies at each site *i*:2.1
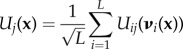
and2.2
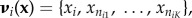
where 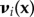 is a vector of amino acids that includes the sequence position *i*, the amino acids of **x** at site *i* along with the amino acids at the *K* neighbours of site *i*, {*n*_*i*1_,…,*n*_*iK*_}. The energy at site *i* is an independent normal random variate for each unique vector of amino acids ***ν***: 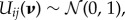 and the factor 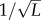 normalizes the variance of the energies with respect to the sequence length *L*. The epitope's energy landscape thus consists of a different random energy at each site, for each unique combination of amino acids at the site along with its epistatically interacting sites.

To facilitate comparison with data, we rescale the energies to affinities with *F*_*j*_ = e^*a*−*bU*_*j*_^ [48]. As described in §3(a), parameters *a* and *b* are chosen to yield mean binding affinities of approximately 10^4^–10^5^ in the naive pool and approximately 10^7^–10^8^ after 30 rounds of affinity maturation [19]. The ruggedness of the energy landscape is controlled by the B-cell sequence length *L*, the number of interaction partners for each site *K* and the alphabet size *A* from which sequences are generated. The value of *K*, which indirectly measures the degree of epistasis, is not well known [50]. We chose an alphabet size of 5, which has been repeatedly identified to efficiently maximize prediction of protein folding with reduced alphabets [53–56], and which has been used in similar NK models [49,57].

### (b) Creation of the naive B-cell repertoire and initiation of the germinal centre response

At the start of an exposure, *N*_G,0_ GCs are each seeded with *N*_B,0_ naive B cells, referred to as B-cell founders [58]. To select founders, a random epitope is chosen, and a cell is randomly generated with an epitope-specific affinity in the top fraction *f* of all possible sequences. This method assumes a fraction *f* of naive B cells bind an antigen above some threshold affinity [59]. Affinity maturation occurs in discrete rounds. The B-cell population increases by a factor of four each round (two divisions per cell) until reaching the maximum GC population size, *N*_B_ [60]. Cells in each new round *d* are generated by copying cells from the previous round with mutation rate *μ* per site, allowing multiple mutations in a single replication event. Each cell in the GC can produce up to four cells in the next round. Actual growth is regulated by relative affinity as described in §2(c).

### (c) Affinity maturation within germinal centres

At the start of every round in a GC, the effective affinity *E_j_* of each B cell to an epitope *j* is adjusted for potential epitope masking by antibodies. Although many antibodies could compete with B cells, we use an approximation:2.3
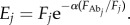


Here, *F_j_* is the intrinsic affinity of the B cell to epitope *j*, 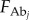 is the affinity of the masking Ab, and *α* is a constant governing the strength of competition between the masking Ab and the BCRs (electronic supplementary material, figure S1). The masking Ab is the Ab with the maximum effective concentration to epitope *j*,2.4
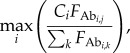
determined by *C_i_*, the concentration of each Ab population *i* (described in §2d) proportional to its affinity, 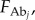 to epitope *j* compared with all of the *k* epitopes. The use of the maximum affinity across epitopes is an approximation made for model simplicity. It can be justified biologically by the assumption that each Ab encounters all epitopes and binds to the one with the highest affinity. We assume that the antigen is presented at a constant rate throughout the GC reaction and is not consumed by B cells or antibodies.

At the end of each round, B cells proliferate proportionally to their maximum effective affinity across all epitopes, *E*_*j*_/∑_*i*_*E*_*i*_, where *E_j_* is the maximum effective affinity of a single B cell, and ∑_*i*_*E*_*i*_ is the sum of the maximum effective affinities of all B cells in the GC. As before, we implicitly assume that a BCR interacts with all epitopes, and that the maximum affinity represents the equilibrium state. B cells with an effective affinity below an absolute threshold *F*_T_ cannot proliferate, and cells without daughter cells are discarded, simulating apoptosis [16,61]. Affinity maturation in each GC continues for 30 total rounds (*R* = 30) or until all effective affinities drop below the absolute threshold, *F*_T_ [62]. The first stopping condition corresponds to the depletion of antigen and is assumed to occur roughly 15 days (at 0.5 days/round) after the initiation of a GC response [63]. The second corresponds to an inability of B cells in the GC to bind antigen that has been masked by abundant high-affinity antibodies.

### (d) Export of cells from the germinal centre and maintenance of the memory population

After each round of affinity maturation, some B cells in each GC are copied and exported as memory or plasma cells. The fraction *m* exported as memory cells decreases linearly with the round number, from *m*_1_ at the outset to *m*_R_ at round 30, which mirrors increased rates of plasma cell export in later rounds. Plasma cells have significantly higher affinities than memory cells after infection [18], although it is unclear if this difference is due to high-affinity thresholds for plasma cell production or greater expansion of high-affinity cells [64]. We assume the former, exporting a fraction *n* of B cells in the GC with affinity above 10^6.5^ as plasma cells [24]. These memory and plasma cells then join their respective global pools.

After leaving the GC, plasma cells immediately begin secreting antibodies that mask epitopes on antigens presented by FDCs. Because we are interested in the affinity maturation in a primary infection over only a few weeks, we fix the initial concentration of exported antibodies, *C*, at unity and ignore decay [23]. Memory B cells do not decay or produce antibodies.

### (e) Software

The model is implemented in C++, with individual B cells and GCs represented as objects. Detailed output, including sequences, intrinsic and effective affinities for each epitope, and Ab concentrations over time, is written to a SQLite database. Because multiple cells can have the same sequence, each sequence is represented once in memory and in the output. For large values of *L* and *A*, generating the full energy landscape is computationally intractable. Instead, we define a deterministic pseudo-random mapping for energies at each locus that is independent of the random number generator used for simulation dynamics. It depends only on an energy seed *s_j_* for the epitope, the sequence position *i* and the neighbour sequence 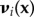.

## 3. Results

### (a) Parametrization and tuning the evolutionary landscape

We developed a stochastic model to simulate affinity maturation to complex antigens. In brief, naive B cells enter GCs and compete to bind to single or multi-epitope antigens. A B cell's sequence and the epitope's energy landscape determine the binding affinity. In each GC, B cells undergo rounds of proliferation, mutation and selection, leading to the loss of low-affinity B cells. Some B cells differentiate into memory or plasma cells and exit the GC. Plasma cells produce antibodies that compete with B cells for antigen by masking epitopes. Each simulation models affinity maturation in one primary infection, and for each set of parameters, we perform 100 replicate simulations.

Empirical estimates exist for many parameters (table 1), but others we fitted by simulation. Consistent with analyses of protein fitness landscapes, we assume few interaction partners, resulting in relatively low ruggedness (*K* = 5) [50]. After 30 rounds of replication, the highest affinity B cells appear at *K* < 6 (electronic supplementary material, figure S2), demonstrating that our landscape permits extensive adaptation. To determine *a* and *b*, which scale energies to affinities by *F*_*j*_ = e^*a*−*bU*_*j*_^, we compared model output to experimental observations. Parameter *a* sets the mean affinity of the energy landscape. The stringency of GC founder selection *f* [59] and *a* together determine the affinity in early rounds (electronic supplementary material, figures S3 and S4), while parameter *b* controls the sensitivity of affinity to changes in energy (electronic supplementary material, figure S3). We fixed the stringency at *f* = 0.001 and chose *a* = 7.0 and *b* = 2.0. In our simulations, B-cell founders thus had affinities between 10^4^ and 10^6^, and over 30 rounds of replication, the mean affinity increased by several orders of magnitude (electronic supplementary material, figure S3) [19].

**Table 1.** Parameter values of the GC model. Description of and values for all parameters used in the model. References are given for values taken from the literature. Other parameter choices are discussed in the text.

symbol	description	default value	reference
*N*_G,0_	initial number of GCs per exposure	100^a^	[60]
*f*	affinity quantile from which to draw GC founders	0.001	[59]
*N*_B,0_	founding B-cell population size (per GC)	5	[58]
*N*_B_	maximum B-cell population (per GC)	1000^a^	[60]
*μ*	mutation rate of B cells during affinity maturation	0.01/site/round	[17]
*D*	maximum number of daughter cells produced per round	4	[16]
*m*_1_	fraction of exported cells becoming memory cells in first round	0.9	text
*m*_*R*_	fraction of exported cells becoming memory cells in round *R*	0.1	text
*n*	fraction of GC cells from which to draw plasma cells	0.1	text
*C*	initial Ab concentration from plasma cells	1	text
*F*_T_	threshold minimum effective affinity for B-cell removal and GC dissolution	10^4.125^	SI
*ε*	duration of round of affinity maturation	0.5 days	[16]
*R*	maximum possible number of rounds of affinity maturation	30	[18]
*q*	number of epitopes per antigen	1	varies
*L*	B-cell sequence length	100	[52]
*K*	number of interaction neighbours	5	text
*A*	size of sequence alphabet	5	[57]
*a*	tuning parameter for energy-affinity mapping	7.0	text
*b*	tuning parameter for energy-affinity mapping	2.0	text
*α*	competition constant for masking Ab and BCR	0.5	text

^a^Because the differences in affinity were not dramatic between 10 GCs × 100 cells/GC and 100 GCs × 1000 cells/GC, we use 10 GCs × 100 cells/GC.

Estimates of the number of GCs and B-cell population sizes per GC vary considerably [60]. We investigated the relationship between the number of GCs, the maximum B-cell population per GC and the extent of affinity maturation (electronic supplementary material, figure S5). Affinity maturation was more sensitive to the maximum B-cell population per GC than to the number of GCs, with larger B-cell population sizes increasing adaptation. The differences were not dramatic, however: as few as 10 GCs, each with a maximum of 100 B cells, gave similar final affinities after 30 rounds as 100 GCs with 1000 cells. Although 2500 B cells per GC yielded even higher final affinities, GCs this large are not common [60]. By default, we simulated with *N*_G,0_ = 10 GCs and *N*_B_ = 100 cells.

### (b) Two types of competition shape affinity maturation

**Figure 2.**
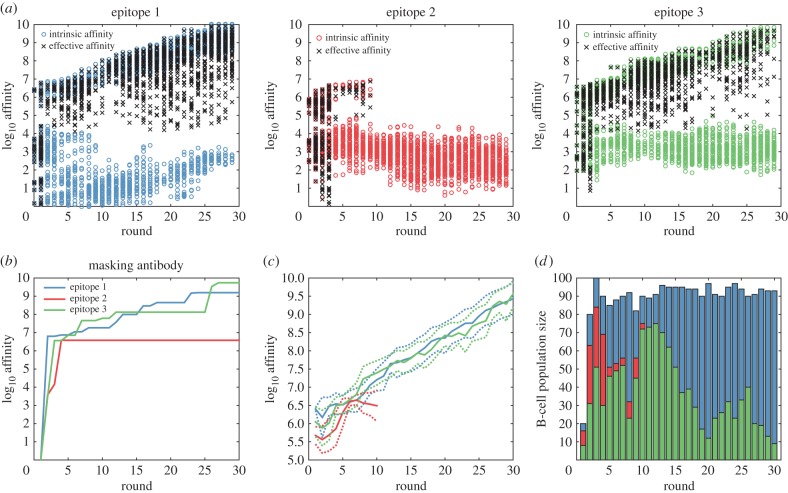
Simulated dynamics of affinity maturation in one GC. (*a*) Intrinsic affinities of the B cells (coloured circles) to each of three epitopes change during the GC reaction. As antibodies begin competing with B cells for antigen, effective affinities (black crosses) diverge from intrinsic affinities. The effective affinity is not pictured when it falls below −1, which occurs when 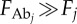. See electronic supplementary material, figure S6, for distributions of affinities in every round. (*b*) The intrinsic affinity of the masking Ab towards each epitope increases during the GC reaction. (*c*) The mean intrinsic affinities of the B cells specific to different epitopes change during the GC reaction. Lines are present only when at least one cell in the GC has highest affinity to that epitope. Dotted lines show 1 s.d. in affinity. See electronic supplementary material, figure S6(*c*), for distributions of affinities including only cells with highest affinity to that epitope, i.e. specificity to that epitope. (*d*) The number of GC B cells specific to each epitope changes during the GC reaction. Results are from a single representative replicate with three epitopes.

Increases in B-cell affinities in our model arise from two types of competition. The first is competition within GCs between evolving lineages. High-affinity B cells have high growth rates, leading to increases in mean affinity over time (figure 2; electronic supplementary material, figure S6). When competition for antigen is removed, so that all B cells have equal fitness, the mean affinity of B cells in a GC declines to the mean of the fitness landscape (electronic supplementary material, figure S7). When few high-affinity naive B cells seed each GC, replicating B cells experience relaxed selection in early rounds. Consequently, mean affinity can initially decline (figure 2*a*; electronic supplementary material, figure S8). Intensifying early competition by increasing the number of founding cells prevents this decline (electronic supplementary material, figure S8*a*). Reducing B cell growth rates and increasing the maximum population size per GC extends the period of relaxed selection and decreases affinities (electronic supplementary material, figure S8*b*,*c*). In most cases, despite strong competition in later rounds, affinity maturation starts to slow as beneficial mutations become rare.

**Figure 3.**
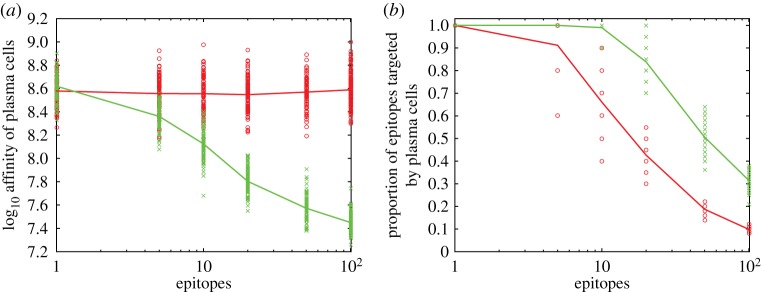
The breadth of affinity-matured B cells declines with the number of epitopes. (*a*) Mean affinities of plasma cells without epitope masking (red line through circles) or with epitope masking (green line through crosses). The calculation of mean affinity only takes into consideration the epitope to which the plasma cells have highest affinity. (*b*) The fraction of epitopes targeted by at least one plasma cell without epitope masking (red line through circles) or with epitope masking (green line through crosses). Targeted epitopes are recognized with affinity greater than 10^6.5^. All replicates assume 10 GCs. Individual points represent means of individual replicates without (red circle) or with (green crosses) epitope masking.

Competition between secreted antibodies and B cells for antigen also influences affinity maturation (figure 2). This competition arises once plasma cells are produced (figure 2) and selects for cells with different specificity (electronic supplementary material, figure S9). Although the mean probability that a daughter cell differs in specificity from its parent peaks early (e.g. with 10 epitopes, at nearly 5% in the third round) (electronic supplementary material, figure S9*a*), and most GCs contain diverged subpopulations through the first few rounds (electronic supplementary material, figure S9*b*), these subpopulations are not selected until much later (electronic supplementary material, figure S9*c*). With few epitopes, epitope masking by antibodies increases the final affinities of plasma cells (Mann–Whitney test, *p* = 0.03); with many epitopes, masking decreases affinities (figure 3*a*). The intuition is that, with many epitopes, B cells not blocked by antibodies outcompete populations that are, leading to rapid changes in specificity in each GC.

### (c) Complex antigens diversify the repertoire but limit its relative breadth

**Figure 4.**
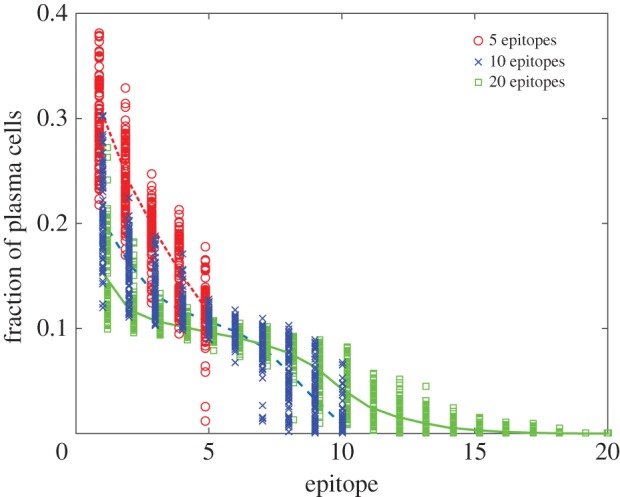
Distributions of plasma cell specificities. The mean frequencies (lines: red short dashed, 5 epitopes; blue long dashed, 10 epitopes; green solid, 20 epitopes) and individual frequencies per replicate (symbols: red circle, 5 epitopes; blue cross, 10 epitopes; green square, 20 epitopes) of populations specific to each epitope are significantly skewed in plasma cells. Each distribution involves 100 replicates each with 10 GCs. Epitopes are sorted from highest abundance to lowest. Points are jittered for visual clarity.

The presence of multiple epitopes promotes the evolution of B cells with different specificities (figure 3*b*). The specificities of both plasma (figure 4) and memory B-cell populations (electronic supplementary material, figure S11) are skewed, with nearly half of the epitopes of a 20-epitope antigen unrecognized. The bias in specificity occurs even when the number of GCs exceeds the number of epitopes (figure 3*b*).

Epitope masking increases the diversity of the plasma and memory repertoires and the fraction of epitopes that are recognized (figure 3*b*). Without epitope masking, high-affinity plasma cells target, on average, 65% of epitopes in a 10-epitope antigen; with masking, they target nearly 100%. This occurs as antibodies block epitopes targeted by local B populations, promoting the growth of B cells with alternative specificities. If B cells bind poorly enough to other epitopes, the GC reaction terminates. GC reactions terminate rarely (less than 2%) before the end of 30 rounds when multiple epitopes are present (electronic supplementary material, figure S10).

B cells with high affinity to more than one epitope could in principle arise. Although GC founder cells have relatively high affinity (for the default value of *f*), epitopes' genotype–phenotype landscapes are independent and uncorrelated. Thus, cross-reactive plasma and memory cells are rare (approx. 0.1% of cells) (electronic supplementary material, figure S12).

### (d) Complex antigens reduce antibody affinities

**Figure 5.**
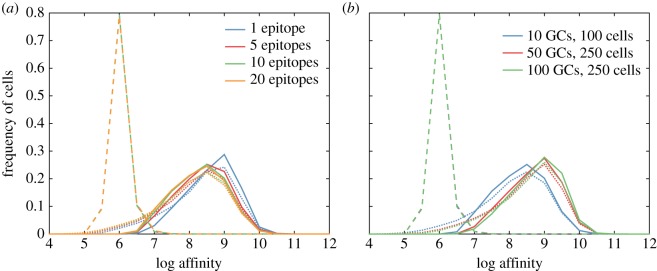
Affinities of founder, memory and plasma populations. (*a*) Increasing the number of epitopes leaves the affinity distributions of GC founder cells (dashed lines) unchanged, while affinities of memory cells (dotted lines) and plasma cells (solid lines) decrease. Simulated distributions use 10 GCs, each with 100 cells. (*b*) With 10 epitopes, increasing the number of GCs or the maximum B-cell population per GC increases affinities of plasma cells (solid lines) but not GC founder cells (dashed lines) or memory cells (dotted lines). Each distribution includes 100 replicates.

Increasing the number of epitopes decreases the frequency of high-affinity plasma cells and memory cells to targeted epitopes (figures 3*a* and 5*a*). The mean affinity of plasma cells declines because the presence of fewer GCs per epitope and fewer B cells per epitope in each GC reduces genetic diversity and compromises adaptation (figure 5*a*; electronic supplementary material, figure S5). Holding the number of epitopes constant while increasing the number of GCs and the maximum B-cell population per GC raises plasma cell affinities (figure 5*b*). Because memory cells are produced continuously but at a decreasing rate during the GC reaction, their population is larger than the plasma cell population, has lower affinity, and is less sensitive to the number of epitopes. Increasing the number of GCs and the maximum population of B cells per GC also raises memory cell affinities, though not to the same extent as for plasma cells (figure 5*b*).

### (e) Stochastic selection of naive B cells determines immunodominant epitopes

**Figure 6.**
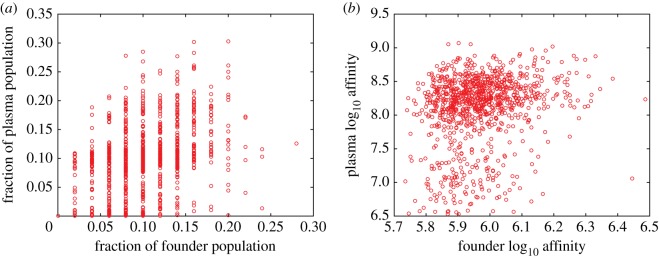
Correlations between GC founder and plasma populations. The fraction of the founder population specific to a particular epitope correlates with (*a*) the fraction of the plasma population specific to the same epitope (Pearson's correlation *ρ* = 0.40, *p* < 10^−5^). The affinity of founder cells correlates with the (*b*) affinity of plasma cells (Pearson's correlation *ρ* = 0.21, *p* < 10^−5^). Graphs show populations to each of 10 epitopes for 100 replicates.

Uneven targeting in primary infections could arise from chance selection of naive B cells, differences in epitopes' immunogenicity, or stochastic mutations. On average, plasma cells have acquired 14 substitutions from germline, and the mean number of mutations increases slightly with the number of epitopes (electronic supplementary material, figure S13). Epitopes do not differ significantly in immunogenicity (mean affinity) in the model, so we examined the correlations between traits of naive B cells and the mature repertoire to infer the impacts of initial conditions and chance mutations. There is a moderate correlation (Pearson's *ρ* = 0.40, *p* < 10^−5^) between the fraction of GC founders with some specificity and the fraction of plasma cells with the same specificity (figure 6*a*). The weaker correlation and high variance between the affinities of GC founders and plasma cells (Pearson's *ρ* = 0.21, *p* < 10^−5^) demonstrate that adaptation is not perfectly commensurate with initial affinity (figure 6*b*).

## 4. Discussion

We developed a model of affinity maturation to understand the basic evolutionary dynamics of the Ab repertoire to a complex antigen. We show that two types of competition, competition between B cells within GCs and competition between secreted antibodies and GC B cells, greatly affect the diversity and affinity of the repertoire. Although complex antigens diversify the repertoire, the mean affinity declines as the number of epitopes increases. This decline occurs because the reductions in the numbers of B cells and GCs per epitope compromise adaptation; reductions in population size and structure should slow adaptation on rugged landscapes [65]. Epitope masking further decreases affinities to multi-epitope antigens, but it increases the probability that the repertoire will target any particular epitope. Thus, it suggests the immune system negotiates a trade-off between high adaptation to few epitopes or lower adaptation to many. Favouring breadth over depth may be a useful strategy when a large fraction of epitopes are non-neutralizing or variable. Our simple model proposes that the dominant specificities in the mature repertoire—the immunodominant clones—are determined mostly by stochastic selection of GC founders. Taken together, the results suggest a mechanism favouring the evolution of antigenically complex pathogens: the repertoires they induce may target fewer epitopes, and the antibodies will have, on average, reduced affinity. It remains to be seen how easily the trade-offs identified here arise in practice, e.g. whether GC B-cell population sizes are highly constrained during co-infections or chronic infections with diverse strains.

Repertoire dynamics underlie the problem of broadly neutralizing antibodies. Despite their importance for natural immunity and vaccine design, the conditions under which broadly cross-reactive neutralizing antibodies [66] evolve are unclear. Two recent models of the repertoires induced by vaccines to malaria [39] and HIV [40] report strikingly different expectations for the evolution of cross-reactive antibodies. Cross-reactive antibodies arose to both variable and conserved antigens in malaria [39] but to only the variant HIV antigens after sequential vaccination [40]. Our model, which is analogous to a single vaccination with a complex antigen or a cocktail of unrelated antigens, did not predict that cross-reactive antibodies would evolve. Similarly, cross-reactive antibodies to HIV after immunization with a cocktail of antigens were rare [40]. The probability of evolving cross-reactive antibodies may be determined by antigenic similarity between epitopes, and any trade-off between specificity and evolvability.

Another important but unresolved dynamic of affinity maturation is selection by T cells. For lack of additional insight, we did not explicitly include T cells in our model. Although B cells clearly compete for limited antigen, recent evidence suggests that signalling from T follicular helper cells is also important [16]. We implicitly represent T cells through affinity-based selection (assuming that high-affinity B cells present more antigen to T cells) and through the maximum GC population size, which depends on the quantity of available antigen and the amount of T-cell help. Other recent work modelled T-cell-dependent selection based on the relative uptake of antigen, which was entirely determined by affinity [40]. This assumption is not inherently different from our own.

The variability between immunodominant and targeted epitopes from run to run underscores the utility of stochastic models in investigating repertoire evolution (figure 2). Even when starting from identical germline sequences, naive B-cell populations diverge in sequence, affinity and even specificity during affinity maturation [67]. Our model suggests that a simple explanation for different patterns of immunodominance is the stochastic selection of naive B cells to found GCs. We speculate that these founder effects fade after multiple infections, allowing repertoires to converge phenotypically. Although we examined a primary infection with variably complex antigens, the model can accommodate multiple exposures to pathogens that vary antigenically over time. Statistically integrating these kinds of models with data may provide quantitative insight into patterns of antigenic variation and vaccination strategies [39,40].

## Supplementary Material

Detailed methods and supplemental figures.

## Data accessibility

The software is available at https://github.com/cobeylab/gcdynamics.
