# Supplementary material for: Trade-offs in antibody repertoires to complex antigens
Source: Philos Trans R Soc Lond B Biol Sci. 2015 Sep 5;370(1676):20140245. doi: 10.1098/rstb.2014.0245 (PMC4528422; doi:10.1098/rstb.2014.0245)
Supplement: Detailed methods and supplemental figures. [file rstb20140245supp1.pdf]

Tradeoffs in antibody repertoires to complex antigens: Supplemental Information

Lauren M. Childs[1,2,*], Edward B. Baskerville[3], Sarah Cobey[3]

1 Center for Communicable Disease Dynamics, Harvard T.H. Chan School of Public Health, Boston, MA, USA
2 Department of Epidemiology, Harvard T.H. Chan School of Public Health, Boston, MA, USA
3 Ecology and Evolution, University of Chicago, Chicago, IL, USA
∗ E-mail: *lchilds@hsph.harvard.edu*

Supplemental Material

Detailed Methods

Calculation of GC dissolution threshold, F_T

As the GC reaction proceeds, epitope masking by antibodies lowers the mean effective affinities of B cells. When no B cells have effective affinity above a threshold, F_T, the GC reaction ends. We set $F_T = 10^{4.125}$. This is determined by competition of antibodies with affinity much above that of the B cell receptor as follows:

$$E_j(\mathbf{x}) = F_j(\mathbf{x}) \exp\left[-\alpha \frac{F_{\mathrm{Ab}_j}}{F_j(\mathbf{x})}\right] < 1, \tag{S1}$$

where α governs the competition between the masking antibody and the B cell receptor; and F_{Ab_j} is the affinity of the masking antibody. The masking antibody is chosen by maximizing over all plasma cells i, and the affinity is normalized over all epitopes k:

$$\max_i \left(\frac{C_i F_{\mathrm{Ab}_{i,j}}}{\sum_k F_{\mathrm{Ab}_{i,k}}} \right). \tag{S2}$$

Simulation Description

This purpose of this section is to provide a description of the model of B-cell affinity maturation, with sufficient detail to implement the simulation in code. Context and justification for the steps can be found in in the Methods. Although the software supports multiple infections, for simplicity this description is limited to a single course of infection in a naive host.

At the beginning of the simulation, an antigen is initialized with q epitopes. $N_{G,0}$ germinal centers are seeded with founder cells. Affinity maturation proceeds through R rounds of growth and selection. At the end of each round, memory and plasma cells are copied and exported from the cells.

Simulation Loop

1. Create an antigen with q epitopes (Antigen Creation).
2. Create $N_{G,0}$ germinal centers (GC Creation).
3. Create an empty list of plasma cells and an empty list of memory cells.
4. Set time $t := 0$.
5. Repeat with round number $r := 1$ to R:

   Repeat with GC number $g := 1$ to N_G, skipping terminated GCs:

   i. Perform one round of affinity maturation in GC g (Affinity Maturation).

   ii. Export plasma cells and memory cells from GC g (Cell Export) and store them on their respective lists.

6. Set time $t := t + \varepsilon$.
7. Remove all plasma cells i where $C_i(t) < C_{\min}$ (Concentration).

Antigen Creation

Repeat for epitope number $j := 1$ to q:

Repeat from site $i := 1$ to L:

Sample distinct neighbors $\mathbf{n}_{ij} = \{n_{ij1}, \dots, n_{ijK}\}$ uniformly randomly from $\{1, \dots, L\} \backslash \{i\}$.

Sample a 32-bit seed s_j to parameterize the mapping between neighbor sequences and energies.

GC Creation

1. Choose a random epitope j.
2. Calculate the energy threshold $U_f = \Phi^{-1}(f)$, where Φ is the cumulative distribution function of the standard normal distribution. The probability that a randomly generated cell will have binding energy lower than U_f to a randomly generated epitope is *a priori* equal to f.
3. Repeat:
   (a) Create a cell with sequence $\mathbf{x}$ drawn uniformly randomly from all possible sequences of length L with alphabet size A.
   (b) Calculate the binding energy $U_j(\mathbf{x})$ of the cell to the epitope (Energy Computation).
   (c) If $U_j(\mathbf{x}) < U_f$, then choose this cell to seed the GC and exit the loop.
4. Initialize a list of cells for the GC with the seed cell.

Affinity Maturation

Affinity maturation in round r proceeds as follows for GC g:

1. Calculate the B cell population size $N_{B,r}$ at the end of this round:

$$N_{B,r} = \min\left(N_B, 4^r\right) \tag{S3}$$

2. Calculate the number of offspring O_i for each existing B cell i in GC g:

   If $N_{B,r} \leq 4^r$, $O_i = 4$ for each cell i.

   Otherwise, initialize $O_i := 0$ for each cell i, and repeat for $j = 1$ to $N_{B,r}$:

   (a) Calculate the probability p_{ij} of choosing existing cell i as a parent for offspring cell j as:

$$p_{ij} \propto \begin{cases} 0 & O_i = 4 \\ \max_k E_k(\mathbf{x}_i) & \text{otherwise} \end{cases} \tag{S4}$$

   where $E_k(\mathbf{x}_i)$ is the effective affinity of parent cell i to epitope k (Affinity Computation).

   (b) Draw parent cell i for offspring cell j proportional to the probability p_{ij}, and set $O_i := O_i + 1$.

3. Initialize an empty list of offspring cells.
4. For each parent cell i:

   For $j = 1$ to O_i:

   (a) Generate an offspring cell sequence $\mathbf{x}_j$. For each site in the sequence k:

   With probability μ, set x_{jk} to a randomly chosen letter of the alphabet such that $x_{jk} \neq x_{ik}$. With probability $1 - \mu$, set $x_{jk} := x_{ik}$.

   (b) Add the offspring cell to the list.

5. Replace the list of current cells in GC g with the list of offspring cells.

Energy Computation

The energy $U_j(\mathbf{x})$ is equal to the sum of energies at each site:

$$U_j(\mathbf{x}) = \frac{1}{\sqrt{L}} \sum_{i=1}^{L} U_{ij}(\boldsymbol{\nu}_i(\mathbf{x})) \tag{S5}$$

$$\boldsymbol{\nu}_i(\mathbf{x}) = \{x_i, x_{n_{ij1}}, \ldots, x_{n_{ijK}}\} \tag{S6}$$

The individual energies U_{ij} are normally distributed via a pseudo-random mapping from the energy seed s_j, the sequence position i, and the neighbor sequence $\boldsymbol{\nu}_i(\mathbf{x})$, using the following computation:

1. Form a byte sequence $b_{ij} := s_j | i | \boldsymbol{\nu}_i(\mathbf{x})$, using four bytes for s_j, two bytes for i, and $K+1$ bytes for the neighbor sequence $\boldsymbol{\nu}_i(\mathbf{x})$, one for each amino acid.
2. Calculate $\text{sha1}_{ij} := \text{SHA1}(b_{ij})$, where SHA1 is the SHA-1 cryptographic hash function [1].
3. Set $u = (U+1)/2^{32}, v = (V+1)/2^{32}$, where U is the first four bytes of sha1_{ij} and V is the next four bytes, and thus u, v are to a discrete approximation uniformly distributed on $(0, 1]$.
4. Set $U_{ij} := \sqrt{-2 \ln u} \cos(2\pi v)$, so that U_{ij} is normally distributed according to the Box-Muller transform [2].

Affinity Computation

The intrinsic affinity $F_j(\mathbf{x})$ of a cell with sequence $\mathbf{x}$ to epitope j is equal to

$$F_j(\mathbf{x}) = \exp\left[a - bU_j(\mathbf{x})\right]. \tag{S7}$$

The effective affinity $E_j(\mathbf{x})$ is equal to

$$E_j(\mathbf{x}) = F_j(\mathbf{x}) \exp\left[-\alpha \frac{F_{\text{Ab}_j}}{F_j(\mathbf{x})}\right], \tag{S8}$$

where α governs the competition between the masking antibody and the B cell receptor, and F_{Ab_j} is the affinity of the masking antibody. The masking antibody is chosen by maximizing over all plasma cells i, and the affinity is normalized over all epitopes k:

$$\max_i \left(\frac{C_i F_{\text{Ab}_{i,j}}}{\sum_k F_{\text{Ab}_{i,k}}} \right). \tag{S9}$$

Cell Export

Memory and plasma cells are chosen for export from GC g in round r as follows:

1. For each cell i in GC g:
   (a) With probability $m_r = m_1 + \frac{r-1}{R-1}(m_R - m_1)$, add a copy of cell i to the list of memory cells.
   (b) If $\max_j E_j(\mathbf{x}_i) > E_{\text{plasma}}$, with probability p add a copy of cell i to the list of memory cells with initial concentration C.

Concentration

The concentration $C_i(t)$ of plasma cell i at time t is given by

$$C_i(t) = C \exp\left[\delta(t - t_i)\right] \tag{S10}$$

where t_i is the time at which the plasma cell was exported and δ is the decay of the antibody concentration, which we assume is 0 during a primary infection.

References

1. PUB F. Secure Hash Standard (SHS). 2012;.
2. Box GE, Muller ME. A note on the generation of random normal deviates. The Annals of Mathematical Statistics. 1958;(29):610–611.

Supplemental Figures

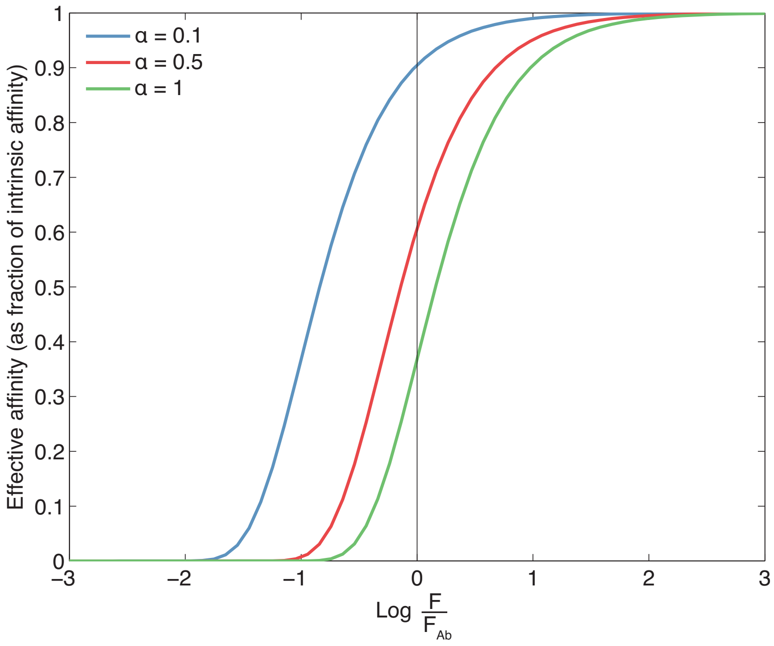

Figure S1. Effective affinity. The effective affinity function increases sharply as the affinity of the B cell receptor, F, approaches the affinity of the masking antibody, F_{Ab}. The relative effectiveness of the masking antibody is controlled by the parameter α.

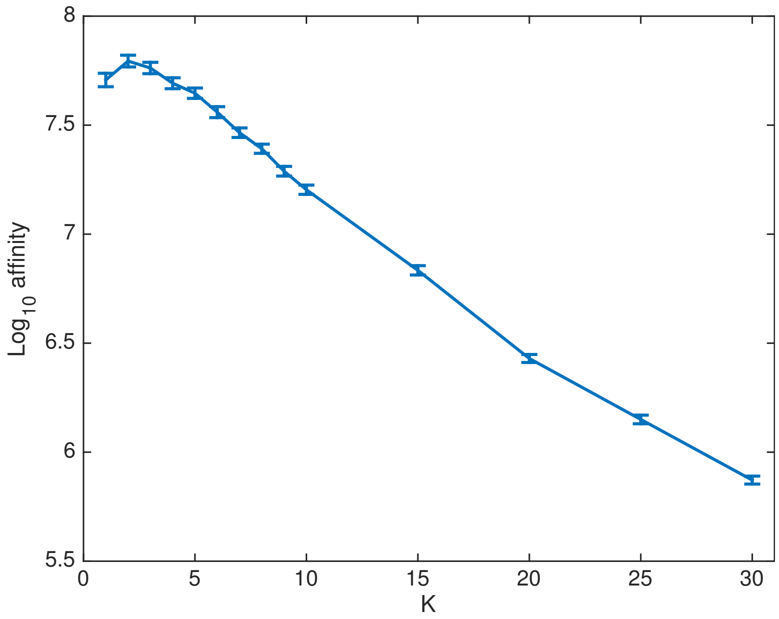

Figure S2. Number of neighbors, K. The affinity achieved to a single epitope after 30 rounds of affinity maturation changes with the number of ineraction neighbors, K. Bars represent standard error of the mean for 100 replicates.

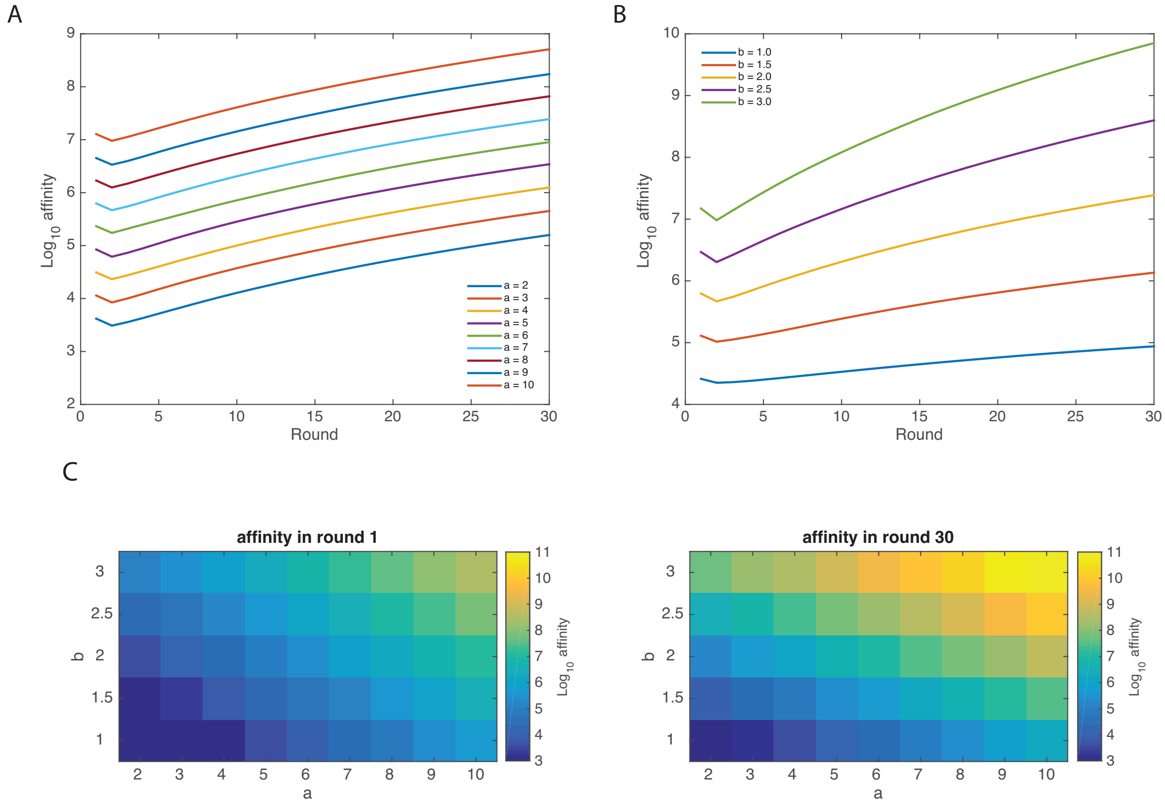

Figure S3. Optimization of model parameters mapping energy to affinity. The values of a (left) and b (right) in $F_j = e^{a-bU_j}$ determine the mean and extent of affinity maturation during the GC reaction. **(A)** The mean affinity achieved is determined by the value of a with $b = 2.0$. **(B)** The variance of the level of affinity achieved is determined by b with $a = 7.0$. **(C)** When a and b are varied, they both contribute to the initial and final affinity. All simulations follow affinity maturation to a single epitope. In **(A)-(B)**, solid lines show the mean and dotted lines the standard error of the mean of 100 replicates.

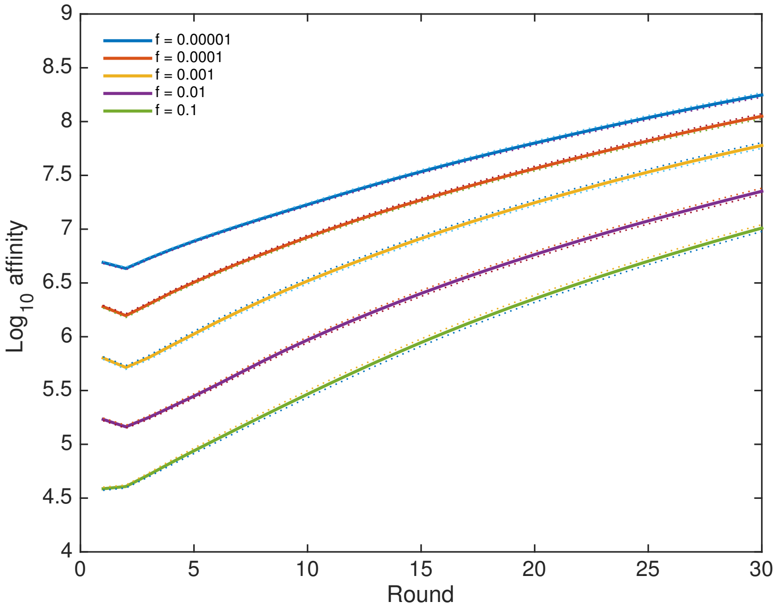

Figure S4. Affinity threshold for naive cell activation. The starting affinity and dynamics of affinity during the GC reaction are affected by the top fraction f of naive cells from which GC founders are selected. Solid lines show the mean and dotted lines the standard error of the mean of 100 replicates.

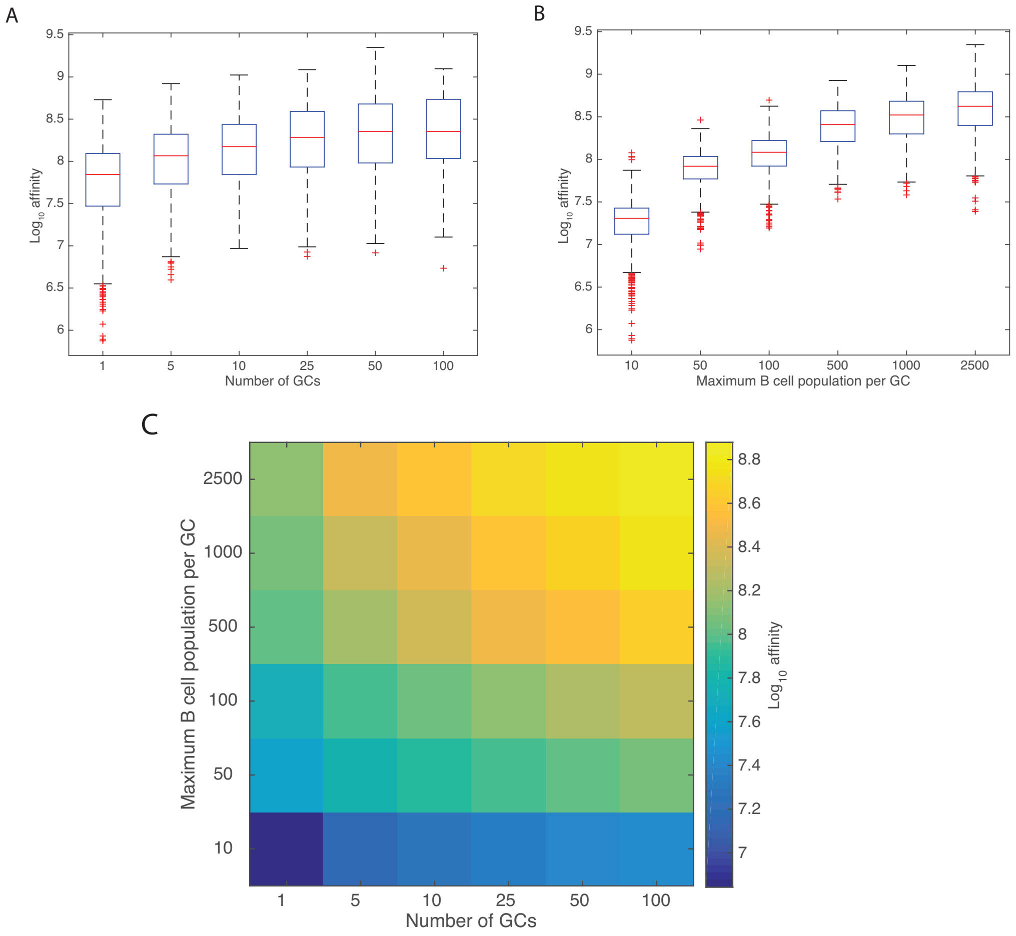

Figure S5. Final affinity as a function of GC size and number. Final affinities to a single epitope increase slightly with the number of GCs **(A)** and maximum B cell population sizes **(B)**. Varying both jointly show greater sensitivity to the maximum GC B cell population size **(C)**. In **(A)**, the maximum GC B cell population size includes 10, 50, 100, 500, 1000, and 2500 cells for each number of GCs. In **(B)**, number of GCs includes 1, 5, 10, 25, 50 and 100 for each maximum GC population size.

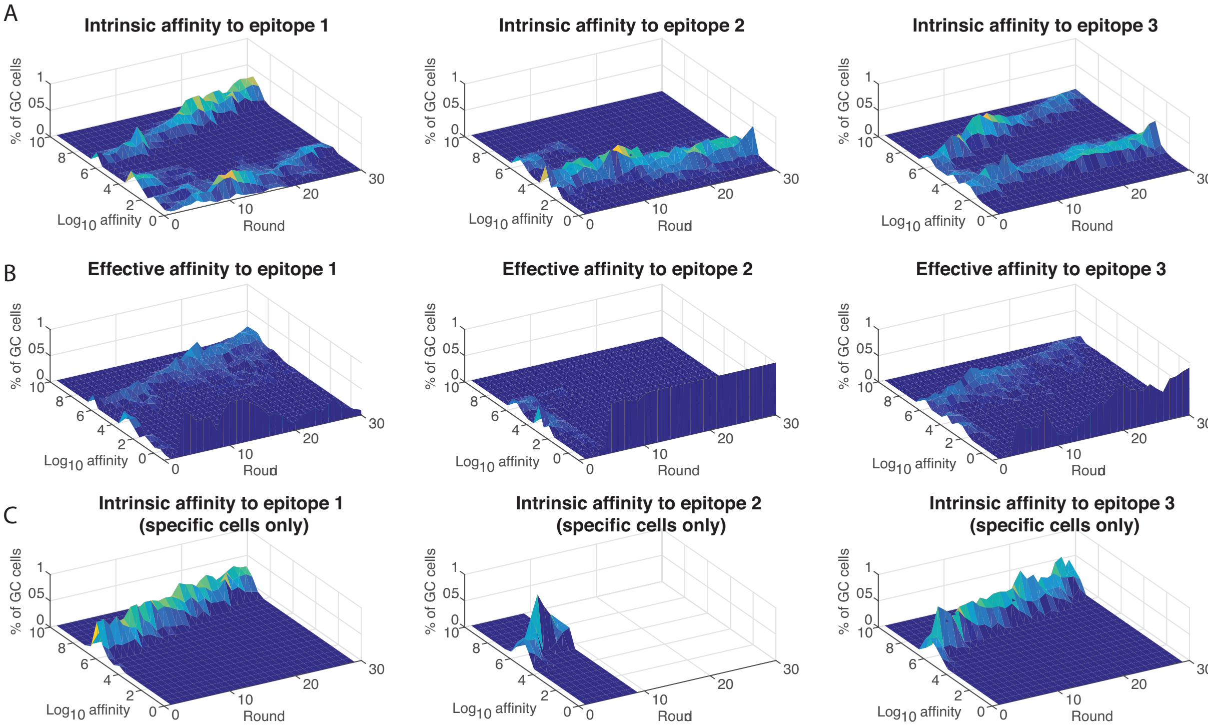

Figure S6. Simulated dynamics of affinity maturation in one GC. (A) The distribution of intrinsic affinities of the B cells to each of three epitopes changes during the GC reaction. **(B)** The distribution of effective affinities of B cells to each of three epitopes changes during the GC reaction. All cells with effective affinity below 0.1 are grouped. **(C)** Distribution of intrinsic affinities of B cells only to the epitope for which they have highest affinity. No cells have highest affinity to epitopes 2 after the initial rounds. Results are from a single representative replicate with three epitopes and a single GC. See Figure 2 for average affinity towards each epitope and the specificity of cells.

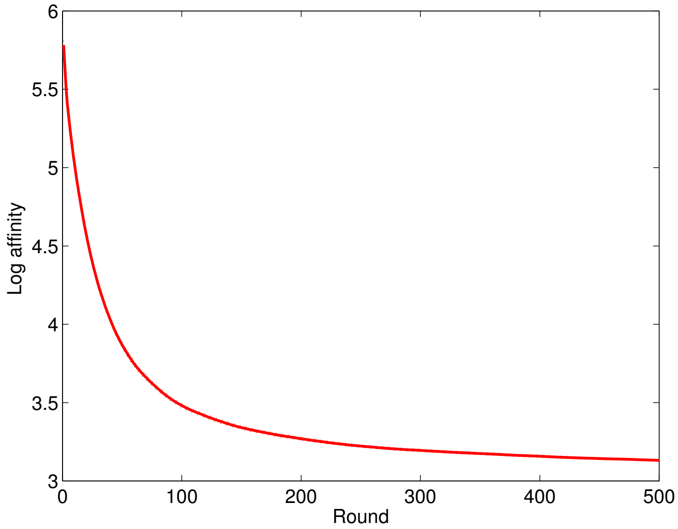

Figure S7. Decoupling B cell affinity and proliferation. When all B cells have equal probabilities of proliferating each round, their affinity declines to approximately the mean of the energy landscape. Solid lines show the mean and dotted lines the standard error of the mean of 100 replicates.

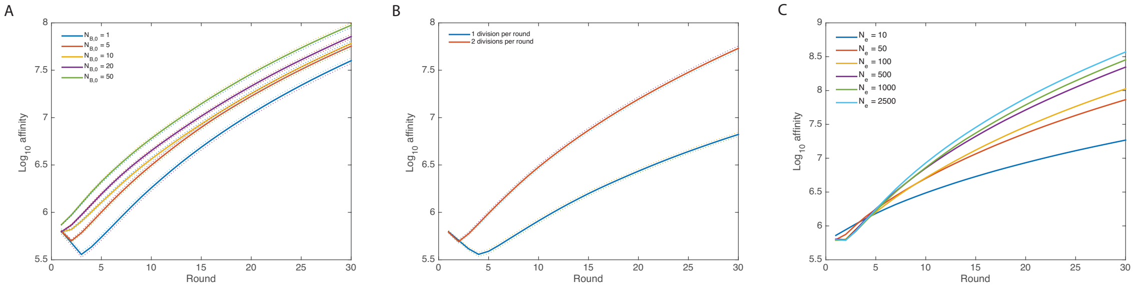

Figure S8. Dynamics of affinity maturation. The decrease in affinity in the early rounds of affinity maturation depends on **(A)** the number of founders per GC, **(B)** the B cell population's growth rate, and **(C)** the maximum population size within a GC. Solid lines show the mean and dotted lines the standard error of the mean of 100 replicates.

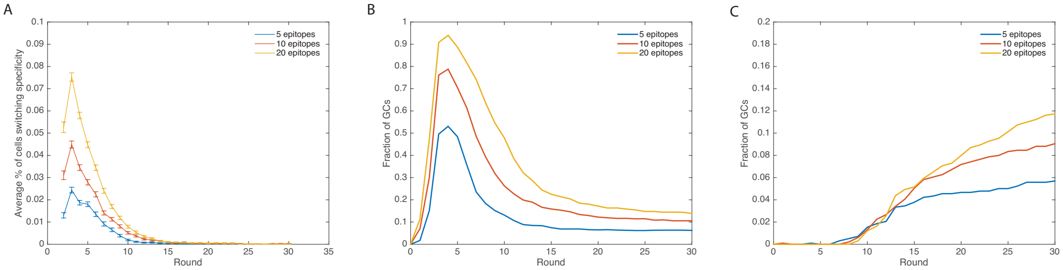

Figure S9. Occurrence of switching epitope of specificity. In each round of the GC reaction, following mutation, a small proportion of cells have different specificity than their parent **(A)**. Most GCs have at least one cell that has switched specificity from the founding cell **(B)**, especially during the early rounds of the GC reaction. The number of GCs where all cells switch specificity from the founder cell is low **(C)**. Each distribution involves 100 replicates each with 10 GCs starting from a single founding B cell.

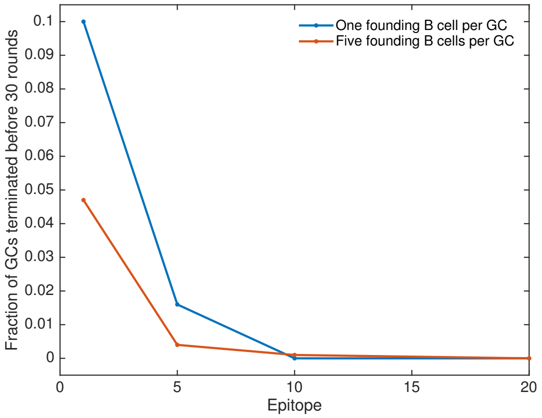

Figure S10. Frequency of GC termination. Termination of GCs prior to 30 rounds is low in the presence of multiple epitopes, particularly when starting from five founding B cells. Each points involves 100 replicates each with 10 GCs.

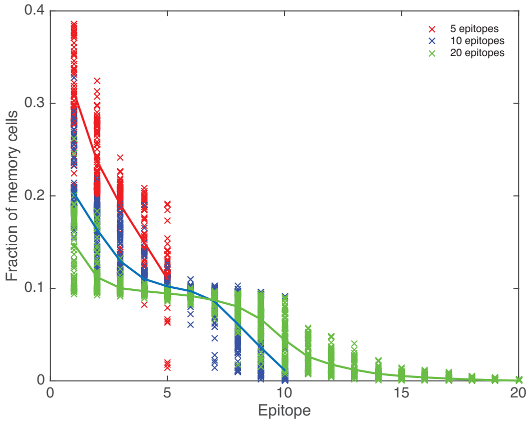

Figure S11. Distributions of memory cell specificities. The mean frequencies (solid lines) and individual frequencies per replicate (color x) of populations specific to each epitope are significantly skewed in memory cells. Each distribution involves 100 replicates each with 10 GCs. Epitopes are sorted from highest abundance to lowest.

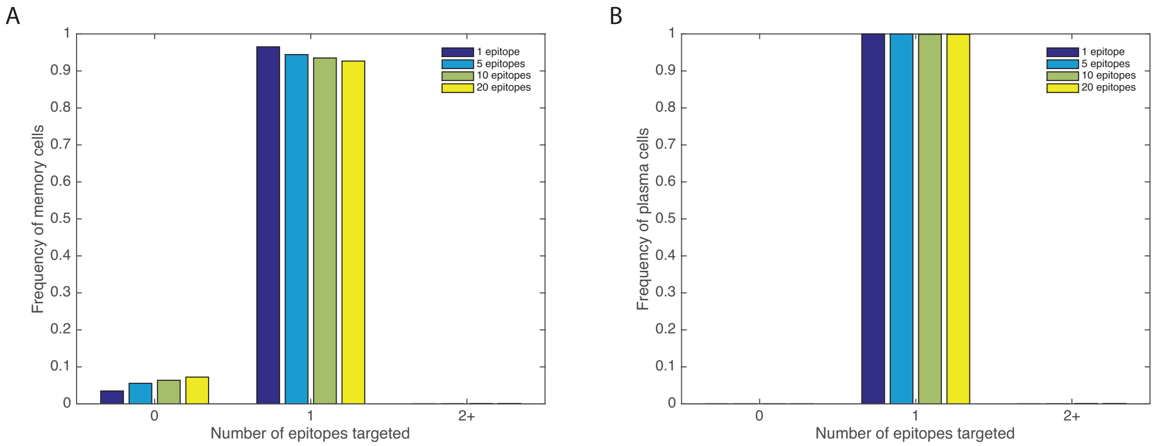

Figure S12. Occurrence of cross-reactive antibodies. The frequency of memory **(A)** or plasma **(B)** B cells that target one or more epitopes. Targeting is determined by affinity greater than $10^{6.5}$, which is the threshold for plasma cells to be exported from the GC.

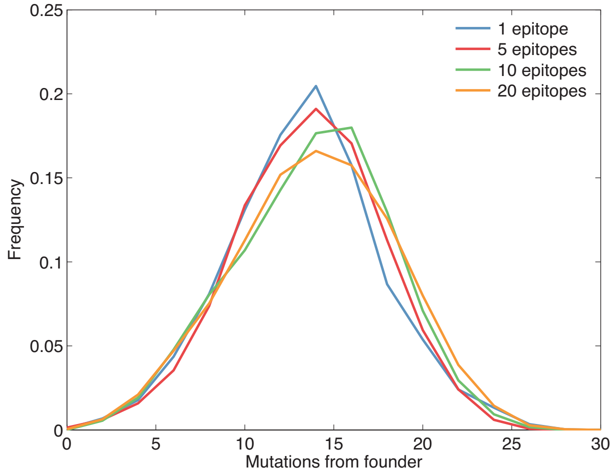

Figure S13. Distributions of mutations from germ line in plasma cells. The mean number of mutations from the founder population of each GC to the plasma cell population only increases slightly with the inclusion of more epitopes. Each distribution involves 100 replicates each with 10 GCs.
